# Modulation of Chemokine Activity for Enhanced Angiogenesis and Tissue Regeneration in Chronic Wounds

**DOI:** 10.3390/ijms27073189

**Published:** 2026-03-31

**Authors:** Ganesh Nideesh Adit, Kavyashree Srikanth, Kannan Harithpriya, Kumar Ganesan, Kunka Mohanram Ramkumar

**Affiliations:** 1 Department of Biotechnology, School of Bioengineering, SRM Institute of Science and Technology, Kattankulathur, Chennai 603203, Tamil Nadu, India; ng3446@srmist.edu.in (G.N.A.); ks8440@srmist.edu.in (K.S.); khp.2201@gmail.com (K.H.); 2 School of Chinese Medicine, Li Ka Shing Faculty of Medicine, The University of Hong Kong, Hong Kong SAR, China; kumarg@hku.hk

**Keywords:** chemokines, wound healing, chronic wounds, angiogenesis, GPCR signaling

## Abstract

Chronic non-healing wounds, prevalent in diabetic and vascular diseases, arise from dysregulated chemokine signaling that disrupts angiogenesis, immune coordination, and tissue remodeling. This review synthesizes current knowledge on chemokine biology in wound repair, with a focus on their spatiotemporal regulation across the hemostasis, inflammation, proliferation, and remodeling phases. We detail chemokine classification (CC, CXC, CX3C, and C families), receptor interactions, and downstream pathways, including G protein-dependent and β-arrestin-biased mechanisms. Furthermore, we evaluate emerging therapeutic strategies, including neutralizing antibodies, receptor antagonists, engineered chemokines, and biomaterial-based delivery systems designed to restore chemokine gradient integrity and promote healing. Recent advances in structural biology and protein engineering are highlighted as enabling the design of biased ligands and multi-target inhibitors to overcome chemokine redundancy. The review concludes that precision modulation of chemokine networks offers a promising translational framework to redirect chronic inflammation toward regenerative healing, thereby addressing a significant unmet clinical need in chronic wound management.

## 1. Introduction

Wound healing is a vital biological process that restores the integrity and function of tissues after injury. It involves a series of overlapping and coordinated stages, including hemostasis, inflammation, proliferation, and tissue remodeling [1]. Each phase relies on the precise recruitment of specific cell types and signaling molecules to the site of injury. Disruption of this coordination can delay or impair healing, often resulting in chronic wounds or excessive scar formation. Understanding the molecular players that control each stage is therefore essential for developing new therapies for impaired healing conditions such as diabetic ulcers, venous ulcers, or surgical complications [2]. Among the key molecular regulators of wound repair are chemokines. Chemokines are a family of small, secreted proteins best known for their ability to attract and direct the movement of cells [3]. They are produced by a wide range of cells, including keratinocytes, fibroblasts, endothelial cells, and immune cells, in response to injury. By creating gradients within the damaged tissue, chemokines guide neutrophils, monocytes, lymphocytes, fibroblasts, and endothelial progenitors toward the wound site. This cell recruitment is crucial for clearing debris and pathogens, releasing growth factors, forming new tissue, and establishing new blood vessels [4].

Chemokines are generally grouped into four main families, CC, CXC, CX3C, and XC, based on the arrangement of conserved cysteine residues in their structure [5,6]. In the context of wound healing, certain chemokines such as CCL2 (MCP-1) and CXCL8 (IL-8) are especially important in the early inflammatory phase for recruiting neutrophils and monocytes. Others, like CXCL12 (SDF-1), promote angiogenesis and the recruitment of progenitor cells during the proliferative phase [7]. Dysregulated chemokine profiles are a hallmark of chronic non-healing wounds, especially in people with diabetes or vascular disease. These observations have led to growing interest in chemokines as potential therapeutic targets and biomarkers for wound repair [8].

While previous reviews have cataloged chemokine functions in wound repair, this article provides an updated synthesis with a dedicated focus on biased signaling mechanisms (G protein vs. β-arrestin pathways) and their therapeutic implications. Furthermore, we emphasize advanced biomaterial-based delivery systems engineered to spatially and temporally control chemokine gradients—a cutting-edge approach not comprehensively covered in earlier reviews. By integrating molecular insights with translational advances in antibody design, receptor antagonism, and engineered chemokines, this work aims to establish a precision medicine framework for targeting chemokine networks in chronic wounds. This review summarizes the current understanding of chemokine involvement in the different stages of wound healing. It highlights how specific chemokines influence cell recruitment, angiogenesis, and tissue remodeling, and discusses the consequences of chemokine dysregulation in chronic wounds. By outlining these mechanisms, the article aims to provide a framework for future research and therapeutic development focused on modulating chemokine pathways to improve healing outcomes.

## 2. Chemokine Classification and Function

Chemokines are grouped according to the arrangement of their conserved cysteine residues. Based on this feature, they are divided into four main subfamilies: CXC chemokines, CC chemokines, CX3C chemokines, and C chemokines. Table 1 provides a concise overview of these families, highlighting representative members, their primary receptors, and their functional contributions to healing.

### 2.1. Cxc Chemokines

CXC chemokines are small, secreted proteins with important roles in immune responses, inflammation, and cancer progression through leukocyte activation. They are characterized by two conserved cysteine residues separated by one amino acid at the N-terminus [9]. According to the presence or absence of a glutamic acid–leucine–arginine (ELR) motif immediately before the first cysteine residue, they are further divided into ELR-positive (angiogenic) and ELR-negative (angiostatic) subgroups [10]. ELR-positive chemokines promote angiogenesis. They attract neutrophils to inflamed tissue and support tissue repair. Members of this group include CXCL1, CXCL2, CXCL3, CXCL5, CXCL6, CXCL7, CXCL8 and CXCL15. ELR+ chemokines such as CXCL1 and CXCL8 stimulate endothelial cell migration and proliferation, which is crucial for blood vessel formation during wound healing [11,12]. By recruiting neutrophils and other immune cells, they regulate inflammation during the initial stages of wound repair [13]. In contrast, ELR-negative chemokines lack the ELR motif and have angiostatic properties. They inhibit new blood vessel formation, a process that must be tightly controlled during wound healing. These chemokines act on cells expressing CXCR1 and CXCR2 and are produced by various cell types in response to pro-inflammatory cytokines such as IL-1β and TNF-α [14]. An example is CXCL10, which blocks endothelial cell recruitment and angiogenesis, thereby restraining excessive vascular growth during wound repair [15].

### 2.2. Cc Chemokines

CC chemokines have two adjacent cysteine residues. This group contains 27 distinct chemokines because CCL9 and CCL10 refer to the same molecule [16]. They signal through 10 different receptors (CCR1–CCR10) [15]. In wound healing, CC chemokines are key for recruiting monocytes and macrophages to the injured tissue [17]. For example, CCL2 controls monocyte/macrophage migration, whereas CCL5 attracts T cells, macrophages, eosinophils, and basophils to sites of inflammation [18]. As healing progresses, CC chemokines also attract cells that suppress inflammation, including regulatory T cells and anti-inflammatory macrophage subsets, shifting the wound environment from inflammatory to reparative [17]. This transition prevents chronic inflammation and supports normal healing. Beyond immune cells, CC chemokines act on keratinocytes and endothelial cells, promoting epithelialization, tissue remodeling, and angiogenesis to complete wound closure [19].

### 2.3. Cx3c Chemokines

The CX3C chemokine family has a single member, fractalkine (CX3CL1) [20]. CX3CL1 and its receptor CX3CR1 are abundantly expressed at wound sites, where they recruit macrophages and fibroblasts. This recruitment stimulates the production of angiogenic mediators essential for healing. Loss of CX3CR1 delays wound closure, as shown by reduced macrophage infiltration and lower vascular endothelial growth factor (VEGF) levels, both critical for collagen deposition and neovascularization [21].

### 2.4. C Chemokines

The C chemokine subfamily contains two members, XCL1 and XCL2. These chemokines attract lymphocytes to inflammatory sites and also recruit macrophages and neutrophils to wounds. Their actions aid in debris removal and support the healing process [4].

## 3. Chemokine Receptors and Signaling Pathways

Chemokines exert their biological effects by binding to specific receptors on the cell surface. These receptors belong to a large superfamily of serpentine proteins [22]. Most chemokines signal through G protein-coupled receptors (GPCRs) to coordinate cell migration. Chemokine receptors are structurally dynamic, and their signaling efficiency depends on precise ligand–receptor interactions [23]. Chemokines bind to their respective receptors and initiate cellular responses that are essential for immune surveillance and inflammatory processes [24].

### 3.1. Cc Chemokine Receptor Family (Ccr)

The CC chemokine receptor (CCR) family comprises ten members (CCR1–CCR10), each mediating the effects of C-C chemokines on distinct immune cell populations. CCR1 binds MIP-1α, MIP-1β, RANTES, and MCP-1, recruiting lymphocytes, monocytes, eosinophils, and basophils, and also interacts with a cytomegalovirus gene product, indicating a role in viral immunity [25]. CCR2, expressed mainly on monocytes but also on dendritic, NK, and T cells, encodes two MCP-1 receptors (CCR2A and CCR2B) and binds MCP-1–4, regulating macrophage trafficking in chronic inflammation such as atherosclerosis and multiple sclerosis [25,26,27]. CCR3 regulates eosinophil migration [28]. CCR4, expressed on activated Th2 cells, binds TARC and MDC to guide T cells to the skin vasculature [29,30,31,32]. CCR5 functions as a chemokine receptor and HIV-1 coreceptor; its ligands include gp120 from M-tropic HIV-1 strains and modified RANTES proteins (Met-RANTES, AOP-RANTES) that antagonize CCR5 and reduce HIV infection [33,34,35,36]. CCR6 binds MIP-3α (LARC) and is upregulated in psoriasis, where keratinocytes colocalize with T lymphocytes [37,38]. CCR7, originally the orphan receptor EBI-1/BLR-2, binds ELC and SLC to direct T and dendritic cell trafficking [30,39,40,41,42,43,44]. CCR8, first known as TER1/ChemR1/CKR-L1, binds I-309, is highly expressed in the thymus, spleen, NK, T, and Th2 cells, and also serves as a coreceptor for various HIV-1 strains [45,46,47]. CCR9, abundant in small-intestinal CD4+ and CD8+ T cells, binds TECK to attract dendritic cells, thymocytes, and macrophages and is critical for gastrointestinal immune responses [48,49]. Finally, CCR10 binds CCL27 (CTACK/Eskine) to activate GPR2 (renamed CCR10) and is highly expressed in the testis, small intestine and keratinocytes, mediating calcium mobilization, mucosal immunity, and skin inflammatory responses such as psoriasis and atopic dermatitis [50,51,52,53].

### 3.2. Cxc Chemokine Receptor Family

The CXC chemokine receptor (CXCR) family comprises receptors CXCR1–CXCR6, which mediate the effects of CXC chemokines on diverse immune and non-immune cells. CXCR1 was identified as one of two IL-8 receptors after high-affinity IL-8 binding sites were discovered on neutrophils [54,55]. Although a CXCR1 gene has not been identified in mice, CXCR1 and CXCR2 likely arose from a common ancestral gene [56]. CXCR2, the other IL-8 receptor, binds both IL-8 and MGSA and is highly expressed in neutrophils, monocytes, and CNS cells such as Purkinje cells and fetal neurons [57,58]. CXCR2 knockout mice show impaired neutrophil mobilization, lymphadenopathy, and splenomegaly [21,59]; CXCR2 also supports B-cell expansion and neutrophil development, and the selective antagonist SB-225002 effectively blocks CXCR2-mediated neutrophil chemotaxis [60]. CXCR3, initially identified as orphan receptor GPR9, is mainly expressed on IL-2-activated T lymphocytes [22,61]. CXCR4, a highly conserved GPCR, was cloned as an orphan receptor before being identified as an HIV-1 coreceptor and ligand for SDF-1 [62]. CXCR5, originally BLR1, is expressed in secondary lymphoid organs and mature B-cell lymphomas; its ligand BCA-1 induces chemotaxis and Ca^2+^ mobilization in murine pre-B and human B lymphocytes but not T cells, monocytes or neutrophils [63,64,65]. Finally, CXCR6, formerly the orphan receptor STRL33/BONZO, is activated by CXCL16 [66]. It facilitates leukocyte adhesion and interacts with the soluble shed form of CXCL16 to regulate leukocyte migration [67].

### 3.3. Cx3c Chemokine Receptor Family

The CX3C chemokine receptor family consists of a single receptor, CX3CR1, which binds fractalkine (CX3CL1), a potent and highly specific chemotactic agonist originally known as V28 before being renamed CX3CR1. This receptor is expressed on monocytes, neutrophils, and T lymphocytes [68] and plays important roles in both inflammation and neuroprotection. In glomerulonephritis, fractalkine expression increases in the glomerular endothelium and CX3CR1 expression rises in infiltrating leukocytes. Administration of anti-CX3CR1 antibodies reduces leukocyte infiltration, prevents crescent formation, and improves renal function [69].

### 3.4. C Chemokine Receptor Family

The C chemokine receptor family contains a single receptor, XCR1, which binds lymphotactin (XCL1), a chemokine lacking the typical cysteine arrangement of other chemokines. XCR1 is predominantly expressed on a specialized subset of dendritic cells known as cross-presenting dendritic cells (cDC1s), and the XCL1–XCR1 axis is crucial for enabling these cells to cross-present antigens and thereby activate cytotoxic T cells and natural killer (NK) cells during antiviral and anti-tumor immune responses [70]. Lymphotactin specifically promotes migration of cells expressing the orphan receptor GPR5 in a chemotactic manner that is inhibited by pertussis toxin, indicating involvement of a Gα-type G protein. It also triggers intracellular calcium mobilization in GPR5-expressing L1.2 cells, with effective mutual cross-desensitization [71].

### 3.5. Signaling Pathways

Chemokine receptors use complex, sometimes biased signaling, and each receptor–ligand interaction can activate different intracellular pathways. Consequently, chemokine-driven cell migration is orchestrated through a network of precisely regulated and selectively biased signaling events [23]. In general, these receptors transduce signals via two major routes: the classical G protein-dependent pathway and the β-arrestin-dependent (G protein-independent) pathway, and the balance between them varies depending on the ligand, receptor, and cell type, a phenomenon referred to as “signaling bias” [72].

## 4. G Protein-Dependent Signaling

Upon ligand binding, the receptor undergoes a conformational change that enables coupling to heterotrimeric G proteins composed of α, β and γ subunits. These GTPases, associated with seven-transmembrane GPCRs, exchange GDP for GTP, leading to dissociation of the G protein into Gα and Gβγ subunits, which then modulate diverse effector proteins [73,74]. Gα subunits are grouped into four families, Gαs, Gαi/o, Gαq and Gα12/13, each linked to specific downstream effectors. Chemokine receptors primarily couple with Gαi, inhibiting adenylate cyclase, lowering cyclic AMP, and reducing protein kinase A activity [75]. Gαi-dependent pathways can also activate Src, leading to ERK1/2 phosphorylation through the MAPK cascade, and stimulate PI3K to produce PIP3, which facilitates actin polymerization and directional cell movement [76,77]. Meanwhile, Gβγ subunits directly activate PI3K to engage small GTPases such as Rac and Rho for cytoskeletal remodeling and chemotaxis [78]. Gβγ also stimulates phospholipase C-β (PLCβ), generating IP3 and diacylglycerol from PIP2; IP3 then mobilizes Ca^2+^ from the endoplasmic reticulum, amplifying intracellular calcium signals critical for migration.

### G Protein-Independent Signaling

Active-state receptors phosphorylated by GPCR kinases (GRKs) recruit β-arrestins, which displace G proteins to desensitize and internalize the receptor [79]. Beyond desensitization, β-arrestins serve as scaffolds for downstream signaling molecules such as Src and MAPK components, enabling G protein-independent pathways [80]. These pathways regulate key cellular functions, including chemotaxis, apoptosis, and granule exocytosis [81]. Typically, β-arrestin-dependent MAPKs remain in the cytosol to act on cytoskeletal substrates, whereas G protein-activated MAPKs translocate to the nucleus to influence gene expression [82,83]. In the CXCL12/CXCR4 axis, β-arrestin 2 is essential for lymphocyte chemotaxis, as shown by impaired migration in β-arrestin 2-deficient mice and CXCR4-transfected cells with low β-arrestin levels [84]. β-arrestins also modulate chemotaxis driven by other receptors such as CXCR1–3 and CCR5 [81]. This interplay between G protein-dependent and β-arrestin-dependent pathways highlights the complexity of chemokine receptor signaling and its central role in immune cell migration, cytoskeletal dynamics and overall cellular responses.

## 5. Specific Contributions of Chemokines to Different Phases of Wound Healing

Chemokines also stimulate the migration and proliferation of fibroblasts, keratinocytes, and endothelial cells, thereby supporting re-epithelialization, granulation tissue formation, and neovascularization. Through these combined actions, cell recruitment, angiogenesis, inflammation control, and cell migration, chemokines form an essential signaling network that coordinates tissue restoration and scar formation during wound healing [4].

The orchestrated contribution of specific chemokines across each phase of wound healing is essential for successful tissue repair. As summarized in Table 2, chemokines such as CXCL8 and CCL2 dominate the early inflammatory response by recruiting neutrophils and monocytes, while later phases are characterized by CXCL12 and CCL21, which support angiogenesis, fibroblast activity, and matrix remodeling. This phase-specific expression highlights the need for precise temporal modulation of chemokine signaling; dysregulation at any stage can lead to impaired healing or chronic inflammation. Understanding these dynamic roles provides a framework for developing stage-targeted therapeutic interventions.

The primary goal of hemostasis is to stop bleeding and form a stable clot. During this stage, chemokines contribute to platelet activation, aggregation, and the formation of a provisional matrix to seal the injury site [98]. Platelets release chemokines such as CCL2, CCL4, and CXCL8, which recruit immune cells to the injury site. Specifically, CCL2 binds to CCR2 on peripheral monocytes, promoting their migration into damaged tissue, which is critical for debris and pathogen clearance [85]. During platelet activation, CXCL4 (Platelet Factor 4, PF4) is released from α-granules to inhibit angiogenesis [86] and has been shown to suppress hematopoiesis and collagenase activity [99,100]. CXCL12 activates CXCR4 on platelets, inducing aggregation and thrombosis via the PI3K and Bruton’s tyrosine kinase (BTK) signaling pathways, independent of classical chemokine migratory activity [87]. CXCL12 secreted by mesenchymal stem cells also promotes cell migration and homing to the injury site [4]. The CXCL12–CXCR4 interaction plays a central role in platelet activation and aggregation, while CCL2 and CCL5 enhance endothelial adhesiveness and leukocyte recruitment [101].

Chemokines play a central role in the inflammatory phase of wound healing by orchestrating the recruitment and activity of leukocytes, which are essential for initiating tissue repair. The primary influx of inflammatory cells is driven by CXCL8, CXCL1, and CXCL2, secreted by platelet α-granules [88]. Neutrophils phagocytose debris and release chemokines such as CCL2, CCL3, and CCL5, which recruit macrophages [102]. A study conducted by Shirmer et al. designed a dressing engineered to bind and sequester multiple pro-inflammatory chemokines, primarily CXCL8 (Interleukin-8), CCL2 (MCP-1), and CXCL1, which contribute to the excessive recruitment of neutrophils and monocytes in chronic wounds.

During the first week post-injury, CC chemokines, including CCL1, CCL2, CCL3, CCL4, CCL5, and CCL7, chemoattract macrophages, resulting in their significant accumulation at the wound site [90]. CXC chemokines such as CXCL1, CXCL2, CXCL5, CXCL7, CXCL8, and CXCL12 directly promote angiogenesis [19]. Chemokines Mig and IP-10 contribute to lymphocyte recruitment around day 4, marking a shift toward adaptive immunity [103]. CXCL10 and CXCL11 are essential for keratinocyte migration during re-epithelialization [91]. Macrophages transition from an M1 pro-inflammatory phenotype to an M2 pro-repair phenotype, resulting in decreased inflammatory markers such as TNF-α and NF-κB, while increasing TGF-β, VEGF, and bFGF production. This phenotypic switch supports angiogenesis, cellular proliferation, migration, and extracellular matrix (ECM) remodeling, setting the stage for the proliferation phase [104].

### 5.1. Chemokine Contribution to the Proliferation Phase

CCL21 and CCL2 promote wound healing by enhancing angiogenesis and recruiting inflammatory cells that release growth factors and cytokines necessary for tissue repair [4]. CCL2 additionally attracts monocytes that differentiate into macrophages, which secrete growth factors to support angiogenesis and extracellular matrix (ECM) formation, while promoting epithelialization and collagen synthesis. Similarly, CCL3 recruits macrophages and supports angiogenic processes, with elevated expression during this phase enhancing cellular signaling for wound repair [85].

CXCL1 and CXCL8 directly stimulate angiogenesis by attracting endothelial cells, contributing to early neovascularization that supplies oxygen and nutrients to proliferating cells [91]. Other chemokines, including CCL5, CCL15, CCL22, CCL28, and XCL1, promote fibroblast proliferation and migration, critical for new tissue formation. Specifically, CCL28 and CCL22 enhance fibroblast activity and oral wound healing by increasing IL-6 and hepatocyte growth factor (HGF) secretion [92]. Chemokines CXCL1–3, CXCL5–8, and their receptors (CXCR1 and CXCR2) further support angiogenesis to nourish newly forming tissue [92]. In re-epithelialization, basal keratinocytes express CXCL10 and CXCL11, and in CXCR3 knockout mice, wound closure and basement membrane regeneration are delayed, highlighting the importance of these chemokines [94].

### 5.2. Chemokine Contribution to the Remodeling Phase

CCL2 continues to recruit monocytes to the wound site, maintaining macrophage presence, which is vital for tissue remodeling and repair. Persistent CCL2 expression, as observed in diabetic wounds, contributes to prolonged macrophage retention and delayed healing [89]. CCL21 aids in the recruitment of T cells and dendritic cells, influencing fibroblast activity and collagen remodeling, while modulating inflammation to affect scar quality [19]. CXCL12 and its receptor CXCR4 recruit progenitor cells and regulate angiogenesis, promoting cell migration and homing required for tissue repair during remodeling [4]. Additionally, CXCL1–3 and CXCL5–8, through their receptors CXCR1 and CXCR2, regulate angiogenesis and keratinocyte proliferation, supporting epidermal regeneration and new vessel formation [92]. CXCL11 (IP-9) and CXCL10 mediate epidermal–dermal communication. A study conducted by Restivo et al. showed that CXCL12 plasmid reduced the healing period to 23 days compared to 55 days in diabetic wounds. 

**Figure 1:**
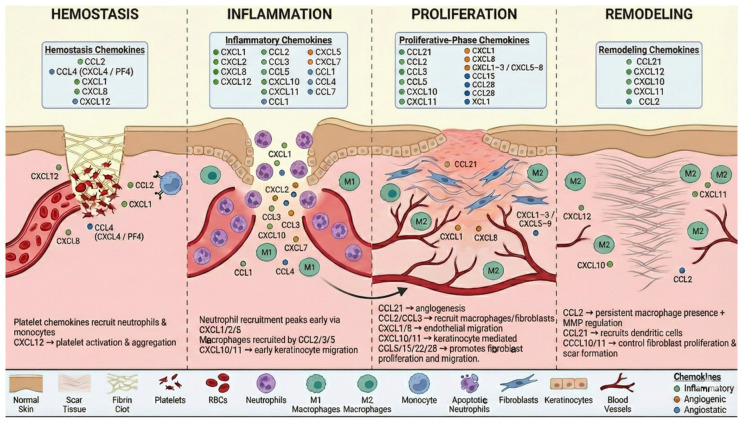
Role of Chemokine in the Four Phases of Wound Healing. Platelet-derived chemokines are involved in clot formation by recruiting neutrophils and monocytes, whereas a few chemokines drive neutrophil influx and M1 macrophage recruitment. Fibroblast- and keratinocyte-derived chemokines aid M2 polarization, neovascularization, fibroblast proliferation and matrix remodeling.

Chemokines also influence matrix metalloproteinases (MMPs) and their inhibitors (TIMPs), balancing ECM degradation and synthesis. For example, CCL2 enhances MMP-12 production by fibroblasts, facilitating collagen turnover [105], while CCL3, CCL4, and CCL5 stimulate MMP-9 secretion by lymphocytes [96]. CXCL10 activates µ-calpain, cleaving β3 integrin, leading to endothelial cell dissociation and subsequent cell death [97]. The coordinated temporal expression of chemokines drives cellular recruitment and functional shifts across the wound healing cascade, as illustrated in Figure 1.

## 6. Therapeutic Strategies Targeting the Chemokine Pathway for Wound Healing

Impaired wound healing leading to chronic wounds is a significant clinical challenge, highlighting the need for novel and effective therapeutic approaches. Over the years, the expression levels of numerous chemokines and their receptors have been recognized as critical determinants in the inflammatory phase of wound healing [7]. Therapeutic strategies designed to target specific inflammatory cells via chemokine modulation are particularly promising for chronic wounds, where persistent inflammation impairs repair [7,8]. Alterations in the physicochemical properties of biomaterials can influence immune responses, and investigating their effects on chemokine expression across different tissues and cell types may facilitate the development of innovative therapies.

Both natural and synthetic biomaterials can be engineered to release specific chemokines or modulate their signaling pathways [85,106]. Among natural biomaterials, glycosaminoglycans (GAGs) play a critical role in extracellular matrix (ECM) interactions and influence chemokine signaling through electrostatic interactions [107]. Variants such as heparan sulfate, hyaluronic acid, chondroitin sulfate, dermatan sulfate, and keratan sulfate have been studied for their ability to modulate inflammatory and angiogenic pathways. For example, heparan sulfate, with its high anionic charge density, attracts pro-inflammatory chemokines like CCL2 and CXCL8, whereas hyaluronic acid can moderate chemokine sequestration, promoting angiogenesis without excessive inflammation [108].

A wide array of natural, synthetic, and hybrid biomaterials has been engineered to modulate chemokine activity and improve wound repair (summarized in Table 3). These materials function through distinct mechanisms—such as sequestering inflammatory chemokines, delivering pro-regenerative chemokines in a controlled manner, or providing structural scaffolds that guide cellular infiltration and tissue organization. Natural polymers like chitosan and alginate are prized for their immunomodulatory properties, while synthetic systems such as PEG and PLGA offer tunable mechanical and release kinetics. Hybrid designs, such as StarPEG–heparin hydrogels, combine the advantages of both classes to precisely control chemokine bioavailability. The strategic application of these biomaterials can shift the wound microenvironment from chronic inflammation toward productive healing.

### 6.1. Natural Biomaterials

Chemical modifications, such as reducing N-acetylation or introducing N-carboxymethyl groups, enhance its therapeutic potential by promoting macrophage polarization toward the M2 anti-inflammatory phenotype and regulating fibroblast activity to mitigate inflammation. Chitosan-based materials can also deliver bioactive compounds, such as CXCL12, to enhance angiogenesis and wound closure in diabetic models. Advanced formulations like succinyl-chitosan combined with dextran and chitosan–genipin scaffolds demonstrate reduced CXCL8 secretion and improved tissue regeneration [110].

Calcium-free alginate upregulates the CXCR7–CXCL12 axis, promoting keratinocyte proliferation and accelerating wound closure [116]. Oxidized alginate gels downregulate inflammatory chemokines while enhancing proliferative-phase markers such as CXCL4 and CCL12, making alginates suitable for both chronic and acute wound applications [112].

Silk fibroin, derived from Bombyx mori, is a biocompatible material that supports angiogenesis and reduces scar formation through controlled release of chemokines such as CXCL1 and CXCL2 [4]. Non-woven fibroin scaffolds enhance pro-angiogenic signaling during the proliferative phase, while gelatin-fibroin microparticles sustain chemokine expression to maintain beneficial inflammatory responses. Fibroin’s versatility allows it to serve both as a scaffold and a carrier for therapeutic agents. Beyond biomaterial-based delivery, several pharmacological and biological strategies are being developed to target chemokine pathways in chronic wounds (Table 4). These include small-molecule receptor antagonists, neutralizing antibodies, engineered chemokines, and pathway modulators such as TLR3 agonists. Each approach aims to correct specific dysregulations—whether by blocking excessive neutrophil recruitment, promoting macrophage polarization, enhancing angiogenesis, or resolving persistent inflammation. Importantly, combination therapies that target multiple nodes within the chemokine network are emerging as a promising strategy to overcome pathway redundancy and improve healing outcomes in complex chronic wounds.

### 6.2. Synthetic Biomaterials

Polyethylene glycol (PEG) hydrogels, for example, can be engineered with precise control over stiffness and cross-linking to modulate immune responses. Loosely crosslinked PEG hydrogels reduce pro-inflammatory chemokines such as CXCL1, while PEG-diacrylate gels (sIPN) enhance epithelialization and improve wound healing. Advanced formulations incorporating hyperbranched poly-L-lysine and MnO_2_ nanosheets further suppress inflammation and promote tissue repair [4,7]. Poly(lactic-co-glycolic acid) (PLGA) is widely used for its biodegradability and polymer compatibility. Electrospun PLGA fibers deliver growth factors such as PDGF-BB, stimulating fibroblast proliferation and angiogenesis, while PLGA nanoparticles serve as efficient drug delivery vehicles for wound care [114]. Poly(methacrylic acid) (PMAA), traditionally industrial, has demonstrated pro-angiogenic properties, supporting tissue regeneration, skin grafting in rats, and wound healing in diabetic mice [115].

Chitosan-hyaluronan membranes provide scaffolds for adipose-derived stem cells, enhancing angiogenesis and collagen deposition [116]. Similarly, chitosan-PVA hydrogels crosslinked with genipin allow sustained release of CXCL12, significantly accelerating wound healing in diabetic and healthy models [117]. In addition to biomaterials, small-molecule inhibitors targeting chemokine receptors offer a strategy to regulate inflammation during wound healing [7,8].

## 7. Crosstalk Between Inflammatory and Angiogenic Signals: Role of Chemokines

Inflammation and angiogenesis are closely linked biological processes that share overlapping mediators and signaling pathways. Chemokines are crucial for this crosstalk as they coordinate leukocyte recruitment and regulate endothelial activation, proliferation, and migration. Their regulation ensures that inflammation initiates tissue repair and angiogenesis sustain tissue regeneration, but dysregulated chemokine signaling contributes to pathological outcomes such as chronic wounds, cancer, and autoimmune disease [120,121].

### Chemokines as Dual Regulators

Chemokines exert dual, opposing functions in angiogenesis, depending on their classification. ELR+CXC chemokines like CXCL1 and CXCL8 are potent angiogenic factors whose activation promotes endothelial proliferation, migration, and vascular permeability, while also recruiting neutrophils that secrete pro-angiogenic mediators [122,123]. Conversely, ELR–chemokines such as CXCL9, CXCL10, and CXCL11 inhibit endothelial proliferation and survival, functioning as angiostatic signals in diseases, including obesity-associated vascular remodeling [120].

Chemokine receptors, like G-protein-coupled receptors, activate downstream pathways, including PI3K/AKT, ERK1/2, and Src, which regulate survival, cytoskeletal dynamics, and migration. For instance, the CXCR2–Src axis has been shown to directly improve endothelial sprouting and osteogenic–angiogenic coupling [122]. CXCL12–CXCR4 signaling contributes to endothelial progenitor recruitment and tissue regeneration, while also influencing tumor dormancy and reactivation in glioblastoma [124]. These findings emphasize that chemokine activity integrates immune recruitment with endothelial repair processes.

**Figure 2:**
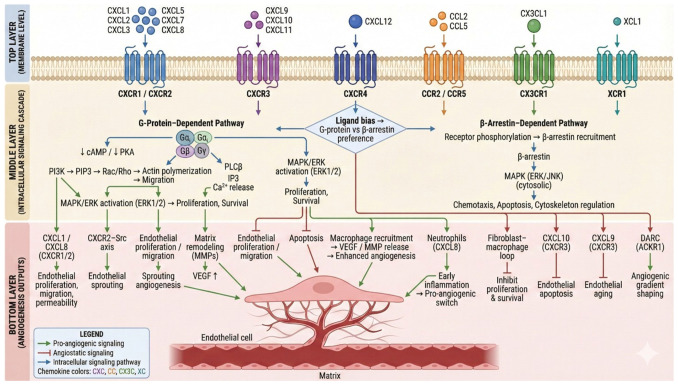
Chemokine Signaling Pathways and their Crosstalk with Angiogenesis. At the endothelial cell membrane, chemokine receptors (CXCR1/2, CXCR3, CXCR4, CCR2/5, CX3CR1, XCR1, DARC) and their ligands activate either β-arrestin-dependent or G-protein-dependent signaling pathways. Pro- and anti-angiogenic outputs, such as sprouting, vessel permeability, inflammatory cell recruitment, and angiogenic gradient formation around endothelial cells, are shaped by downstream cascades (PI3K, Rac/Rho, PLCβ, MAPK/ERK, Src) that control endothelial migration, proliferation, survival, apoptosis, and matrix remodeling.

Chemokines interact directly with classical angiogenic pathways. VEGF stimulates chemokine expression, including CXCL10 and CCL2, in inflammatory microenvironments [125], while chemokines such as CXCL12 and CXCL16 modulate angiogenic growth factor signaling during cellular dormancy transitions [124]. Crosstalk between VEGFR2 and NF-κB also regulates pathological neovascularisation in autoimmune diseases such as Sjögren’s syndrome [126]. Additionally, collagen-IV-mediated signaling has been shown to regulate chemokine production and promote angiogenesis in metastatic sites [127]. In chronic wounds, chemokine-mediated dysfunction between macrophages and fibroblasts under hyperglycemic and LPS-driven conditions impairs angiogenesis and tissue healing [128]. In cancer, chemokines play a key role in preparing metastatic sites and lymphatic endothelial activation [129]. CXCL9-driven endothelial senescence contributes to cardiovascular aging and angiogenic decline [130]. Chemokine receptors activate diverse downstream signaling cascades that integrate immune recruitment with angiogenic programming, as depicted in Figure 2. Crosstalk between chemokine networks and classical angiogenic mediators (e.g., VEGF) further fine-tunes neovascularization. This mechanistic framework highlights the potential for biased ligands and pathway-specific inhibitors to selectively modulate pro-angiogenic or anti-inflammatory outcomes in wound healing.

## 8. Chemokine Receptor Antagonists

Dysregulated chemokine receptor signaling contributes to cancer progression, chronic inflammation, and impaired tissue repair [131]. Structural studies of GPCRs, including CCR5, CCR8, CCR1, CXCR4, and CCR6, have revealed the molecular basis for ligand binding and receptor activation, forming a foundation for therapeutic receptor antagonism [132].

### 8.1. Small-Molecule Antagonists

Small-molecule inhibitors remain one of the most actively explored approaches for chemokine receptor inhibition. CXCR4-targeting drugs have advanced significantly due to detailed mechanistic studies of receptor–ligand interactions. Structural dissection of CXCR4 binding to CXCL12 and hBD-3 demonstrates competing ligands at the same receptor, providing reasons for developing potent antagonists [133]. Such precision molecular design supports the development of next-generation CXCR4-targeting inhibitors capable of modulating migration, metastasis, and inflammatory trafficking. Small-molecule antagonists have also been developed for CCR1, CCR6, CCR8, and CCR2. Cryo-EM structures of G protein–biased CCR1 ligands revealed that Tyr291 plays a critical role in regulating β-arrestin recruitment, a mechanism that can be used to selectively reduce pro-inflammatory signaling without impairing beneficial pathways [132]. CCR6 allosteric antagonists have been structurally characterized and shown to bind distinct inactive conformations, representing a strategy for stabilizing non-signaling states in autoimmune disease conditions [134]. These studies confirm that chemokine receptor antagonists can directly suppress malignancy-associated inflammation and invasive behavior. Furthermore, encoded-library technologies have enabled identification of IL-36 receptor antagonists that reduce inflammatory responses in barrier tissues such as skin [135].

### 8.2. Monoclonal Antibodies

Monoclonal antibodies offer high specificity and sustained antagonism of chemokine receptors. Structural elucidation of CCR8 in complex with an inhibitory monoclonal antibody (mAb1) revealed precise blocking of the orthosteric site, preventing chemokine-induced activation relevant for tumor immunotherapy [136]. Similarly, nanobody-based antagonists have been developed for atypical chemokine receptor 3 (ACKR3), demonstrating selective inhibition of constitutively active or ligand-induced conformations [137]. These engineered biologics broaden the therapeutic possibilities for receptors involved not only in inflammation but also in cancer, fibrosis, and metabolic dysfunction.

### 8.3. Biased Ligands, Allosteric Modulators, and Advanced Antagonist Platforms

Recent studies highlight the advantage of biased signaling modulators over classical antagonists. For example, plerixafor’s biased pharmacology, exhibiting β-arrestin recruitment agonism while antagonizing G protein signaling was shown to be essential for its superior hematopoietic stem cell mobilization efficacy. Chemokine dimerization states can also shape receptor function; CXCL12 dimers alter CXCR4 internalization kinetics and impact leukemic cell migration [138]. Groundbreaking antagonist platforms include CXCR4-targeted nano threads that physically block receptor signaling to inhibit cancer metastasis [139], peptide-functionalized nanoparticles that combine imaging with CXCR4 antagonism [140], and computationally engineered GPCR–peptide signaling complexes that allow precise chemotactic control [141]. CXCR7 antagonism reduces fibrosis in mineralocorticoid-excess models [142], yet CXCR4–CXCL12 dynamics remain critical for tissue repair, immune homeostasis, and even glucocorticoid response in leukemia [143]. Collectively, these studies show that chemokine receptor antagonists including small molecules, antibodies, nanomaterials, and biased ligands, represent a rapidly advancing therapeutic sector. However, successful translation requires consideration of receptor context, compensatory pathways, and structural insights into ligand-specific activation mechanisms.

## 9. Neutralizing Antibodies Targeting Chemokines in Wound Healing

Neutralizing antibodies (nAbs) directed against chemokines represent a precise and increasingly promising strategy to modulate aberrant inflammation in chronic and diabetic wounds. This approach is particularly relevant in wounds where persistent chemokine expression sustains leukocyte infiltration, inhibits angiogenesis, and prevents entry into the proliferative phase of repair.

### 9.1. Neutralizing Antibodies to Pro-Inflammatory Chemokines Improve Healing in Diabetic and Chronic Wounds

Chen et al. (2023) demonstrated that anti-CCL28 antibody administration significantly accelerated wound closure, attenuated NF-κB-dependent inflammatory signaling, reduced macrophage infiltration, and enhanced angiogenesis in diabetic mice [144]. This work establishes that neutralizing hyperactive chemokines can shift the wound environment toward pro-repair conditions. CCL2 neutralization, although extensively studied in cancer models, has clear implications for wound healing. In a metastatic neuroblastoma model, anti-CCL2 antibodies suppressed inflammatory monocyte recruitment and prolonged survival. As CCL2 overexpression similarly contributes to macrophage-driven chronic inflammation in diabetic wounds, these findings support the concept that blocking CCL2 may reduce pathological macrophage burden and restore inflammatory balance. In addition, inhibition of the CCL5/CCL7–CCR1 axis has been shown to promote M2-biased macrophage polarization and reduce tissue-destructive inflammation in osteoarthritis [145], emphasizing that chemokine neutralization can reprogram macrophage phenotypes in tissue injury conditions.

### 9.2. Targeting Excessive Cxc Chemokines: Neutralization of Cxcl8 and Cxcl10

CXC chemokines that disproportionately recruit and activate neutrophils contribute significantly to delayed wound healing. Neutralizing antibody studies revealed that CXCL8-mediated neutrophil activation occurs independently of the atypical receptor CCRL2, demonstrating that direct blocking of CXCL8 is sufficient to reduce excessive neutrophil activation [146]. CXCL10, an interferon-inducible angiostatic chemokine, is frequently elevated in non-healing wounds. In vivo, CXCL10 neutralization requires antibody binding to its GAG-bound form rather than the soluble chemokine alone, as shown by Bonvin et al. 2017 [147]. Effective blocking of CXCL10, therefore, depends on antibodies specifically designed to target matrix-associated chemokines, which are the biologically active form in inflamed tissues.

### 9.3. Autoantibodies to Chemokines Reveal the Biological Impact of Chemokine Neutralization

Chemokine-neutralizing autoantibodies arise naturally following infection and can significantly influence disease progression. Muri et al, identified autoantibodies against chemokines, including CXCL12, CCL21, and CXCL13, in individuals recovering from SARS-CoV-2, with titers correlating with distinct inflammatory and clinical outcomes [148]. These findings provide strong mechanistic support for the idea that chemokine neutralization in vivo can substantially alter leukocyte trafficking, inflammatory resolution, and tissue remodeling. Neutralizing such regenerative chemokines could impair rather than enhance tissue repair, emphasizing the importance of selectively blocking inflammatory chemokines while preserving pro-repair pathways.

### 9.4. Multi-Target Chemokine Network Neutralization Strategies

Given the redundancy of chemokine signaling networks, with multiple chemokines often activating shared receptors, neutralizing a single chemokine may incompletely suppress inflammation. Recent advances using combinatorial saturation mutagenesis (CoSMOS) have yielded engineered multi-chemokine inhibitors capable of neutralizing several inflammatory chemokines simultaneously [149]. Mogamulizumab, an anti-CCR4 monoclonal antibody evaluated in neoadjuvant immunotherapy trials, exemplifies the ability of therapeutic antibodies to redirect immune cell migration [150]. Neutralization of inflammatory chemokines such as CCL28, CCL2, CXCL8, and CXCL10 reduces excessive macrophage and neutrophil infiltration, reduces angiostatic pressure, and supports the transition from persistent inflammation to tissue remodeling [144,146,151]. In contrast, sparing regenerative chemokines such as CXCL12 maintains progenitor cell recruitment, angiogenesis, and epithelial cell migration, processes essential for constructive repair [152,153]. When applied with such pathway-specific precision, chemokine-neutralizing antibodies constitute a powerful therapeutic modality capable of reshaping dysregulated wound environments and promoting effective tissue regeneration.

## 10. Modified Chemokines

Chemokines form a highly interconnected signaling system that precisely regulates leukocyte trafficking, immune cell activation, and inflammatory responses. Their functional redundancy, where multiple chemokines often activate the same receptors, and their context-dependent signaling dynamics pose major challenges for therapeutic targeting. As a result, traditional single-chemokine blocking strategies often fail to achieve robust disease control. Recent advances in structural biology and protein engineering have made it possible to redesign chemokine-binding surfaces or alter receptor–ligand activation properties, enabling strategies capable of overcoming chemokine network redundancy [154].

**Figure 3:**
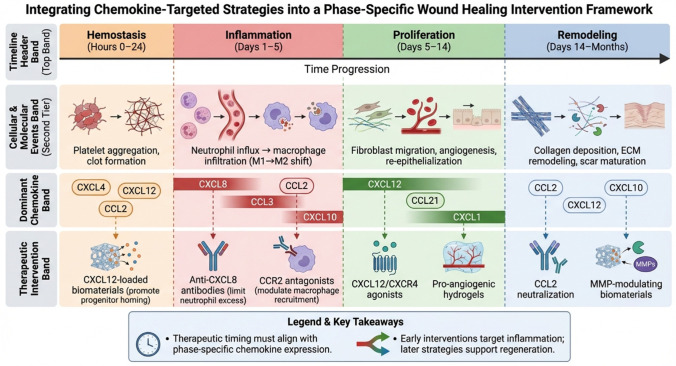
Phase-specific chemokine-targeted intervention framework for wound healing. The diagram illustrates the temporal alignment of therapeutic strategies with the dynamic chemokine and cellular landscape across the four stages of wound repair. The top timeline divides healing into hemostasis (0–24 h), inflammation (days 1–5), proliferation (days 5–14), and remodeling (days 14–months). Cellular events (second tier) depict key processes: platelet aggregation and clot formation, neutrophil and macrophage infiltration, fibroblast and endothelial cell recruitment, and extracellular matrix remodeling. Dominant chemokines (third tier) are shown as expression peaks for each phase (e.g., CXCL8 and CCL2 in inflammation; CXCL12 in proliferation). Therapeutic interventions (bottom tier) are mapped to the phase in which they are most effective: antiplatelet/coagulation modulators and CXCL12-loaded biomaterials in hemostasis; neutrophil-targeting antibodies (anti-CXCL8) and macrophage modulators (CCR2 antagonists) in inflammation; pro-angiogenic factors (CXCL12, VEGF) and fibroblast-activating scaffolds in proliferation; and anti-fibrotic/ECM-modulating agents (CCL2 neutralizers, MMP regulators) in remodeling. Color coding corresponds to each healing phase. This framework emphasizes that therapeutic efficacy depends on precise timing relative to the endogenous chemokine cascade.

Translating chemokine-targeted strategies from bench to bedside requires not only mechanistic insight but also clinical validation. Table 5 summarizes key therapeutic agents under investigation, ranging from repurposed drugs like the CXCR4 antagonist plerixafor to novel biologics such as anti-CCL28 antibodies and engineered evasins. These candidates illustrate diverse modalities—small molecules, monoclonal antibodies, biomaterial-delivered chemokines, and immune modulators—each aiming to correct specific dysregulations in the wound microenvironment. The success of chemokine-based therapeutics hinges not only on target selection but also on precise temporal alignment with the natural progression of wound healing. Figure 3 presents a phase-specific intervention framework that maps recommended therapeutic strategies—from neutralizing antibodies and receptor antagonists to biomaterial-delivered chemokines—onto the dynamic chemokine and cellular landscape of each repair stage. By integrating mechanism with timing, this framework provides a strategic roadmap for translating chemokine biology into staged, intelligent wound therapies.

### 10.1. Engineered Evasins and Synthetic Chemokine-Binding Proteins

Tick evasins have emerged as powerful templates for engineering modified chemokines due to their ability to bind multiple inflammatory chemokines with high-affinity. Devkota et al. [154] structurally characterized subclass A3 evasins and demonstrated that targeted modifications of the chemokine-binding interface can reprogram selectivity across CC and CXC chemokines. Guided by crystal structures and mutational modeling, engineered variants exhibited expanded or redirected chemokine-binding profiles while maintaining stability and inhibitory capacity. This research highlights the feasibility of constructing broad-spectrum chemokine inhibitors capable of reducing complex inflammatory circuits, particularly in diseases where multiple redundant chemokines sustain pathology. Complementing this approach, Vales et al. employed phage display, saturation mutagenesis, and computational docking to identify short peptides with multi-chemokine inhibitory activity. These engineered peptides mimic key binding determinants of evasins but are smaller, synthetically accessible, and adaptable [158]. Their broad-spectrum inhibition of inflammatory chemokines supports their therapeutic potential in settings such as autoimmune disease, transplant rejection, and chronic inflammatory tissue damage. Together, these studies establish engineered chemokine-binding scaffolds as a rapidly expanding class of biologics capable of targeting chemokine redundancy, an essential barrier in chemokine-directed therapies.

### 10.2. Receptor-Biased and Location-Specific Chemokine Modulators

Chemokine signaling is not solely determined by ligand binding; subcellular localization and receptor conformational bias play critical roles in determining signaling outputs. Eiger et al. (2022) demonstrated that biased CXCR3 agonists generate distinct signaling depending on whether receptor activation occurs at the plasma membrane or endosomes [159]. Modified chemokines and engineered agonists could therefore be designed to selectively activate desired signaling cascades such as ERK and β-arrestin without fully recapitulating pro-inflammatory or migratory responses. These insights broaden the conceptual framework for modified chemokines; rather than simply blocking signaling, engineered ligands can reshape the spatial and temporal dimensions of receptor activation.

### 10.3. Structural Modification of Chemokine Regions That Control Receptor Engagement

Advances in structural modeling have revealed that chemokine regions beyond canonical binding motifs can modulate receptor activation. Pinheiro et al. (2025) mapped noncanonical structural elements of CCL25 that control CCR9 activation [160]. Using AI-based predictive modeling combined with mutagenesis, the authors demonstrated that modifications within the chemokine core, rather than the N-terminal receptor-binding motif alone, contributed to biased signaling outputs. Additionally, enzymes such as arylsulfatases and neuraminidases can modify chemokine–receptor interactions by removing key electrostatic groups. Pinheiro et al. (2025) showed that post-translational modification of CCR5 ligands, either by enzymatic desulfation or removal of terminal sialic acids, alters receptor engagement and downstream signaling [160]. These findings illustrate an alternative route for chemokine modification based on biochemical editing rather than sequence engineering [160].

### 10.4. Structural Insights Enabling Rational Chemokine Engineering

Atomic-resolution structures of chemokine–receptor complexes provide critical templates for designing modified ligands. Zhang et al. (2021) solved structures of CCR5 in complex with chemokine MIP-1α and G-protein subunits, identifying distinct binding modes that stabilize receptor activation [161]. Such structures reveal which chemokine residues contribute to activation and inhibition and thereby inform rational engineering of chemokine variants capable of selectively blocking or activating CCR5 [161]. Modified chemokines represent a rapidly advancing frontier in immunomodulatory therapeutics. Structural engineering of chemokine-binding proteins like evasins, generation of synthetic chemokine inhibitory peptides, design of biased or location-specific chemokine agonists, and biochemical modification of chemokine moieties all contribute to a diverse and powerful therapy. These innovations overcome longstanding challenges such as redundancy, compensatory chemokine expression, and off-target inflammation. As structural and computational modeling advances, modified chemokines are shaped to transform therapeutic strategies across cancer immunotherapy, autoimmune disease, infection, and tissue repair.

## 11. Future Directions

Despite significant progress in understanding chemokine biology and the development of neutralizing antibodies and engineered chemokines, substantial knowledge gaps remain in translating these mechanistic insights into effective therapeutic strategies. One key area for future investigation is the development of multi-target approaches capable of overcoming chemokine network redundancy. Advances in structural biology and computational protein engineering are expected to play a central role. Cryo-EM and deep-learning-guided structural modeling have already identified cryptic epitopes on chemokine receptors and viral glycoproteins that are accessible only in certain conformations. Importantly, this may facilitate the generation of selective agonists or antagonists capable of modulating disease-relevant signaling without impairing homeostatic immune function. An additional frontier lies in understanding the temporal dynamics of chemokine signaling during disease progression. Longitudinal profiling of chemokine expression and receptor availability using single-cell transcriptomics, ligand–receptor interaction mapping, and spatial proteomics will help identify areas in which chemokine-targeted interventions are most effective. Such insights may support the development of adaptive or stage-specific intervention strategies. Finally, improved delivery platforms are essential for clinical translation. mRNA-based antibody delivery, nanoparticles, and viral vectors have already demonstrated their potential to enable localized or sustained release of potent neutralizing agents. Applying similar systems to deliver engineered chemokines or chemokine inhibitors may enhance therapeutic precision while minimizing systemic toxicity. Overall, future work must combine molecular engineering, system-level profiling, and innovative delivery strategies to fully harness the therapeutic potential of modified chemokines and neutralizing antibodies.

## 12. Conclusions

Chemokines and their receptors orchestrate inflammation, immunity, and tissue repair through intricate signaling networks. Dysregulation of these pathways underpins chronic non-healing wounds, particularly in diabetic and vascular patients, in which impaired chemokine gradients disrupt angiogenesis, immune coordination, and matrix remodeling. This review has highlighted how advances in structural biology, protein engineering, and biomaterial science are enabling novel therapeutic strategies—from neutralizing antibodies and biased receptor ligands to engineered chemokines and controlled release scaffolds.

Despite these advances, several key challenges must be addressed to translate chemokine-targeted therapies into clinical practice. First, the redundancy and context dependence of chemokine signaling necessitate multi-target approaches, such as bispecific inhibitors or combination therapies, to overcome compensatory pathway activation. Second, temporal precision in intervention is critical; therapies must align with the dynamic phases of wound healing to avoid disrupting beneficial inflammation or delaying repair. Third, patient-specific factors, including comorbidities, microbiome influences, and genetic variations in chemokine receptors, may dictate therapeutic responsiveness and require personalized treatment strategies.

Future research should prioritize the development of smart biomaterials capable of real-time chemokine sensing and release, the clinical validation of biased ligands that selectively promote regenerative over inflammatory signaling, and the integration of multi-omics profiling to identify predictive biomarkers of healing. Additionally, the long-term safety and immunogenicity of engineered chemokines and antibodies must be thoroughly evaluated in preclinical and early-phase trials.

In conclusion, modulating chemokine networks represents a promising frontier in precision wound care. By coupling molecular insight with innovative delivery systems and patient-stratified approaches, next-generation therapies can transform chronic wound management, shifting the paradigm from symptomatic treatment to biologically driven regeneration.

## Figures and Tables

**Table 1:** Chemokine family summary: Key members, receptors, and primary roles in wound healing.

Family	Example Members	Key Receptors	Primary Role in Wound Healing
CXC (ELR+)	CXCL1, CXCL2, CXCL8 (IL-8)	CXCR1, CXCR2	Neutrophil recruitment, angiogenesis, early inflammation
CXC (ELR−)	CXCL9, CXCL10 (IP-10), CXCL11	CXCR3	Angiostasis, lymphocyte recruitment, resolution of inflammation
CC	CCL2 (MCP-1), CCL3 (MIP-1α), CCL5 (RANTES)	CCR1, CCR2, CCR5	Monocyte/macrophage recruitment, T-cell chemotaxis, inflammation resolution, fibrosis modulation
CX3C	CX3CL1 (Fractalkine)	CX3CR1	Macrophage and fibroblast recruitment, vascular integrity, neuro-immune crosstalk
XC	XCL1 (Lymphotactin), XCL2	XCR1	Lymphocyte and dendritic cell recruitment, adaptive immunity priming

**Table 2:** Phase-Specific Roles of Chemokines in Wound Repair and Tissue Remodeling.

Phase of Wound Healing	Chemokines Involved	Source (Cell Types)	Functions	References
Hemostasis	CCL2 (MCP-1), CCL4	Platelets	Recruits monocytes and immune cells; primes inflammation	[85]
	CXCL8 (IL-8), CXCL1	Platelets, Endothelial cells	Initiates neutrophil recruitment and priming; early inflammatory response	[4]
	CXCL4 (PF4)	Platelets	Inhibits angiogenesis; suppresses hematopoiesis and collagenase activity	[86]
	CXCL12 (SDF-1)	Mesenchymal stem cells, Platelets	Activates platelet aggregation; mediates cell migration to the injury site	[87]
Inflammation	CXCL8 (IL-8)	Platelets, Neutrophils	Major neutrophil recruitment; initiates and maintains acute inflammatory response	[88]
	CXCL1, CXCL2	Platelet α-granules, Neutrophils	Promotes neutrophil recruitment; enhances angiogenesis	[19]
	CCL2 (MCP-1)	Neutrophils, Monocytes, Basal epidermis	Monocyte/macrophage recruitment; transition to macrophage response; resolution of inflammation	[89]
	CCL3 (MIP-1α)	Neutrophils, Macrophages	Recruits macrophages, T cells, eosinophils; amplifies inflammation	[85]
	CCL4	Platelets, Macrophages	Macrophage chemoattractant; primes further immune cell influx	[90]
	CCL5 (RANTES)	Neutrophils	Attracts T cells, eosinophils, macrophages; augments inflammatory cell influx	[18]
	CXCL5, CXCL7, CXCL12 (SDF-1)	Platelets, Macrophages, MSC, Endothelial cells	Promotes angiogenesis; recurits neutrophil and progenitor cells	[19]
	CCL1, CCL7	Wound mononuclear cells, Macrophages	Macrophage and monocyte chemoattractant during the initial response	[90]
	CXCL10 (IP-10)	Keratinocytes	Modulates lymphocyte recruitment; limits angiogenesis	[91]
	CXCL11	Keratinocytes	Keratinocyte migration; re-epithelialization	[90]
Proliferation	CCL21	Endothelial cells, Macrophages	Promotes angiogenesis; recruits inflammatory and repair cells	[4]
	CCL3 (MIP-1α), CCL2 (MCP-1)	Macrophages, Fibroblasts	Angiogenesis and tissue regeneration; macrophage recruitment; ECM formation; collagen synthesis	[85]
	CXCL1, CXCL8 (IL-8)	Endothelial cells, Keratinocytes	Endothelial cell recruitment; angiogenesis	[91]
	CCL5, CCL15	Fibroblasts	Fibroblast proliferation, migration; new tissue formation	[92]
	CCL22, CCL28	Oral keratinocytes, Fibroblasts	Enhances fibroblast activity and oral wound healing via IL-6 and HGF	[92]
	XCL1	Keratinocytes, Fibroblasts	Lymphocyte and macrophage recruitment for tissue regeneration	[4]
	CXCL2, CXCL3, CXCL5	Endothelial cells, Fibroblasts	Endothelial cell migration; angiogenesis; keratinocyte proliferation	[93]
	CXCL7	Platelets	Supports new vessel formation	[19]
	CXCL12 (SDF-1)	Mesenchymal stem cells	Progenitor cell recruitment; angiogenesis; regeneration	[4]
	CXCL10 (IP-10), CXCL11	Keratinocytes, Basal epidermis	Modulates wound re-epithelialization; keratinocyte migration and proliferation	[94]
Remodeling	CCL2 (MCP-1)	Macrophages, Fibroblasts	Monocyte/macrophage recruitment for ECM remodeling; prolonged expression linked to chronic wounds	[89]
	CCL21	Endothelial cells, Macrophages	T-cell and dendritic cell recruitment; collagen remodeling; scar quality modulation	[19]
	CXCL12 (SDF-1)	Endothelial cells, Mesenchymal stem cells	Progenitor cell homing; angiogenesis; tissue repair	[4]
	CXCL1, CXCL2, CXCL3, CXCL5	Fibroblasts, Keratinocytes, Endothelial cells	Angiogenesis and keratinocyte proliferation for epidermal regeneration	[93]
	CXCL7	Platelets	Supports new vessel formation	[19]
	CXCL8 (IL-8)	Keratinocytes, Endothelial cells	Angiogenesis/epidermal regeneration	[93]
	CXCL10 (IP-10)	Keratinocytes	Modulates fibroblast and keratinocyte activity; limits fibroblast proliferation; balances remodeling	[95]
	CXCL11	Keratinocytes	Keratinocyte migration, re-epithelialization, scar quality	[95]
	CCL3 (MIP-1α), CCL4, CCL5 (RANTES)	Lymphocytes, Macrophages, Fibroblasts	Stimulates MMP-9 secretion; ECM turnover	[96]
	CXCL10 (IP-10)	Endothelial cells	Activates calpain; cleaves β3 integrin; endothelial dissociation	[97]

**Table 3:** Pharmacological- and Biomaterial-Based Strategies for Modulating Chemokine Pathways in Wound Repair.

Biomaterial	Type	Mechanism of Action	Role in Wound Healing	References
Heparan sulfate	Natural	Attracts pro-inflammatory chemokines (CCL2, CXCL8) via electrostatic interactions	Modulates inflammation by sequestering chemokines at the wound site	[107,108]
Hyaluronic acid		Moderates chemokine sequestration; balances chemokine availability	Promotes angiogenesis without excessive inflammation	[107,108]
Chondroitin sulfate		Modulates chemokine signaling via ECM interactions	Influences inflammatory and angiogenic pathways	[107]
Dermatan sulfate		Modulates chemokine signaling via ECM interactions	Influences inflammatory and angiogenic pathways	[107]
Keratan sulfate		Modulates chemokine signaling via ECM interactions	Influences inflammatory and angiogenic pathways	[107]
Chitosan (modified)		Promotes M2 macrophage polarization; regulates fibroblast activity to reduce inflammatory chemokine secretion	Mitigates inflammation; functions as an immunomodulatory scaffold	[109]
Chitosan-CXCL12 delivery system		Delivers CXCL12; activates CXCR4 for progenitor cell recruitment and angiogenesis	Enhances angiogenesis; accelerates wound closure	[109]
Succinyl-chitosan with dextran		Reduces CXCL8 secretion; modulates inflammation	Decreases neutrophil recruitment; improves regeneration	[110]
Chitosan-genipin scaffolds		Reduces CXCL8 secretion; modulates inflammatory chemokine expression	Improves tissue regeneration; controls inflammation	[110]
Calcium-free alginate		Upregulates CXCR7-CXCL12 axis; enhances keratinocyte proliferation	Promotes keratinocyte proliferation, accelerates closure	[111]
Oxidized alginate gels		Downregulates inflammatory chemokines; enhances CXCL4/CCL12	Modulates chemokine dynamics; transitions wound phases	[112]
Silk fibroin (non-woven scaffolds)		Controlled release of CXCL1; CXCL2 during proliferation	Supports angiogenesis; reduces scarring	[4]
Gelatin-fibroin microparticles		Sustains chemokine expression for beneficial inflammation	Scaffold/carrier for agents; supports controlled inflammation	[113]
Polyethylene glycol (PEG) hydrogels	Synthetic	Reduces CXCL1 via controlled stiffness/crosslinking	Modulates immune response; decreases inflammation	[7]
PEG-diacrylate gels (sIPN)		Modulates chemokine signaling for epithelialization	Improves healing via immune modulation and epithelialization	[4]
PEG with poly-L-lysine/MnO_2_ nanosheets		Suppresses inflammatory chemokine expression	Suppresses inflammation; promotes tissue repair	[4,7]
Polylactic-co-glycolic acid (PLGA) fibers		Delivers growth factors (PDGF-BB) to regulate chemokines and stimulate angiogenesis	Stimulates fibroblast proliferation and angiogenesis	[114]
PLGA nanoparticles		Drug delivery for chemokine modulators	Efficient wound care therapeutic delivery	[114]
Polymethacrylic acid (PMAA)		Pro-angiogenic; modulates chemokine signaling for vascularization	Supports regeneration, skin grafting, and diabetic wound healing	[115]
StarPEG-heparin hydrogels	Hybrid	Heparin binds/sequesters CCL2, CXCL8; PEG enables controlled delivery	Sequesters inflammatory mediators; delivers anti-inflammatory agents	[108]
Chitosan-hyaluronan membranes		Scaffold for stem cells secreting pro-angiogenic chemokines	Enhances angiogenesis and collagen deposition	[116]
Chitosan-PVA hydrogels (genipin crosslinked)		Sustained CXCL12 release; activates CXCR4 for progenitor recruitment/angiogenesis	Accelerates healing via controlled CXCL12 delivery	[117]

**Table 4:** Therapeutic Strategies for Chemokine Modulation in Wound Healing.

Strategy	Target (Molecule/Pathway)	Mechanism of Action	Role in Wound Healing	References
Chemokine receptor antagonists	CCR2, CXCR2, CXCR4, CX3CR1	Block chemokine–receptor binding; inhibit downstream G protein/β-arrestin signaling; reduce leukocyte recruitment	Suppress excessive inflammation; prevent chronic wound formation; promote transition to repair phase	[7,85]
Neutralizing antibodies	CCL2, CXCL8, CXCL10, CX3CL1	Bind and neutralize chemokines; prevent receptor activation and cell migration	Reduce inflammatory cell influx; limit tissue damage; support orderly healing progression	[85,118]
Modified chemokines	CXCL12 (SDF-1), CCL2 (MCP-1)	Engineer chemokines for enhanced stability or altered receptor specificity; boost recruitment of progenitor cells	Enhance angiogenesis; accelerate tissue regeneration; improve closure in chronic wounds	[4,112]
Biomaterial-mediated chemokine delivery	CXCL12, CCL2, CXCL8, CXCL1	Use natural/synthetic/hybrid biomaterials to deliver or sequester chemokines; modulate local gradients and cell influx	Fine-tune inflammation; promote angiogenesis; support proliferation and remodeling	[107,108,109]
Small-molecule inhibitors	Chemokine GPCRs (e.g., CXCR4)	Inhibit receptor signaling (G protein/β-arrestin); block cell migration and activation	Control immune cell recruitment; reduce chronic inflammation; support tissue repair	[7,85]
TLR3 pathway modulation	TLR3/TRIF, downstream chemokines	Activate TLR3 signaling to upregulate chemokines (MIP-2/CXCL2, MCP-1/CCL2, MIP-1α/CCL3); enhance immune response	Accelerate wound closure; promote re-epithelialization, angiogenesis, and ECM remodeling	[118]
Combination therapies	Multiple chemokines/receptors	Target several chemokine pathways simultaneously for synergistic effects	Balance inflammation and repair; improve healing outcomes in complex or chronic wounds	[85,119]

**Table 5:** Clinically investigated and emerging chemokine-targeted therapies for wound healing.

Therapeutic Agent	Target (Chemokine/Receptor)	Type	Development Stage	Proposed Mechanism in Wounds	References
Plerixafor (AMD3100)	CXCR4 antagonist	Small-molecule	Approved (stem cell mobilization); Investigational for wounds	Blocks the CXCL12/CXCR4 axis; may reduce pathological inflammation and enhance progenitor cell recruitment to the wound site.	[85]
Mogamulizumab	CCR4	Monoclonal antibody	Approved (cancer); Repurposing potential	Depletes CCR4+ regulatory T cells and pro-inflammatory lymphocytes; could resolve chronic inflammation in non-healing wounds.	[155]
Anti-CCL28 Antibody	CCL28	Neutralizing antibody	Preclinical (murine models)	Neutralizes overexpressed CCL28 in diabetic wounds; reduces NF-κB signaling, macrophage infiltration, and enhances angiogenesis.	[135]
CXCL12-Loaded Hydrogel (Chitosan-PVA)	CXCL12 delivery	Biomaterial + chemokine	Preclinical/Translational	Sustained, localized release of CXCL12 promotes CXCR4+ cell homing, angiogenesis, and accelerates closure in diabetic models.	[114,156]
Engineered Evasins (A3 subclass)	Broad-spectrum CC/CXC chemokines	Engineered chemokine-binding protein	Preclinical	Broad inhibition of inflammatory chemokines via high-affinity binding; resolves excessive inflammation and may prevent healing impairment.	[145]
Anti-CCL2 Antibody (Carlumab)	CCL2	Neutralizing antibody	Phase II (cancer); wound studies preclinical	Reduces monocyte/macrophage recruitment and M1 polarization; shifts wound microenvironment toward pro-repair states.	[157]
TLR3 Agonists (e.g., Poly(I:C))	TLR3/TRIF pathway	Immune modulator	Preclinical	Activates endogenous chemokine production (e.g., CXCL2, CCL2, CCL3); enhances re-epithelialization and angiogenesis in impaired healing.	[115,117]
Biased CCR1 Ligand	CCR1 (G protein-biased)	Small-molecule	Preclinical	Selectively promotes G protein signaling over β-arrestin recruitment; may reduce fibrosis while preserving beneficial inflammation resolution.	[132]

## Data Availability

No new data were created or analyzed in this study. Data sharing is not included in this article, as it is a comprehensive review of previously published literature. All referenced studies are cited in the reference list, and readers are directed to those sources for the original data.
